# Lobectomy vs total thyroidectomy for unilateral papillary thyroid carcinoma with ipsilateral cervical lymph node metastasis

**DOI:** 10.3389/fendo.2025.1564752

**Published:** 2025-07-24

**Authors:** Shijia Zhang, Kehui Zhou, Jinyu Wang, Ming Zhao, Xiaochun Mao, Jinbiao Shang, Xiabin Lan

**Affiliations:** ^1^ Department of Thyroid Surgery, Zhejiang Cancer Hospital, Hangzhou Institute of Medicine (HIM), Chinese Academy of Sciences, Hangzhou, Zhejiang, China; ^2^ Postgraduate Training Base Alliance of Wenzhou Medical University (Zhejiang Cancer Hospital), Hangzhou, Zhejiang, China; ^3^ Department of Medical Records and Statistics, Zhejiang Cancer Hospital, Hangzhou Institute of Medicine (HIM), Chinese Academy of Sciences, Hangzhou, Zhejiang, China

**Keywords:** unilateral lobectomy, total thyroidectomy, papillary thyroid carcinoma, recurrence-free survival, propensity score matching

## Abstract

**Objective:**

This study aimed to compare the prognosis of unilateral papillary thyroid carcinoma(PTC) patients with ipsilateral cervical lymph node metastasis(IC-LNM) under the treatment of unilateral lobectomy(uLT) vs total thyroidectomy(TT) in order to find out the optimal surgery for these patients without other clinical risk characteristics.

**Methods:**

PTC patients at Zhejiang Cancer Hospital between 2012 and 2022 were retrospectively reviewed. Additionally, a propensity score matching(PSM) was performed on patients treated with uLT or TT. Recurrence-free survival(RFS), overall survival(OS), hospitalization costs, postoperative complications, and other clinical characteristics were analyzed between the two groups.

**Results:**

Ultimately, 682 unilateral PTC patients with IC-LNM were available in the study. After PSM with possible prognostic factors(such as gender, age, primary tumor size, multifocality, extrathyroidal invasion, and T-stage), 225 pairs of patients were available. With a median of 81(5-154) months follow-up, 22 patients(9.8%) in the uLT and 12(5.3%) in the TT recurred. There were no significant differences in 5-year RFS and 5-year OS between uLT and TT groups. However, TT group was significantly correlated with higher risk of transient and permanent hypoparathyroidism, higher levothyroxine doses, longer hospital stays, and higher hospitalization costs than uLT group(p<0.05).

**Conclusions:**

Our study indicated that there were no differences in recurrence and survival between unilateral PTC patients with IC-LNM treated with uLT or TT for the primary tumor. However, uLT group had a lower risk of postoperative complications and a lower hospitalization cost than TT group. Thus, for selected unilateral PTC patients with IC-LNM without other risk features, uLT could be recommended.

## Introduction

Papillary thyroid carcinoma (PTC) accounts for more than 90% of all thyroid cancer cases, and its incidence has increased rapidly in recent years (1, 2). Compared with other malignant tumors, most PTCs have a relatively favorable prognosis, so that the treatment is different from that of other tumors (3, 4). PTC patients commonly have cervical lymph node metastasis, especially central lymph node metastasis, which can reach 20%-50% (5–8). However, the relationship between lymph node metastasis and the prognosis of PTC is still controversial (9–11). Lateral cervical lymph node metastasis is relatively rare compared to central lymph node metastasis, but some studies have shown that lateral cervical lymph node metastasis is associated with increased mortality and recurrence (12–15).

The 2015 American Thyroid Association (ATA) Guidelines recommend near-total or total thyroidectomy (TT) for PTC patients with lateral cervical lymph node metastasis (16). However, TT might increase the risks of recurrent laryngeal nerve injury and transient or permanent hypoparathyroidism (17), while unilateral lobectomy (uLT) could reduce the doses and side effects of postoperative levothyroxine (L-T4) intake, and after uLT, remnant thyroid tissue could produce triiodothyronine (T3) to maintain thyroid hormone balance (18). There were a few studies comparing the prognosis of unilateral PTC patients with ipsilateral cervical lymph node metastasis (IC-LNM; level II-V) treated with uLT and TT, and some studies have shown that there was no significant difference in the recurrence between the two managements (19, 20). Therefore, whether the unilateral PTC patients with IC-LNM should be treated with TT deserves further study.

In this study, our aim was to evaluate the risks and benefits of postoperative complications, prognosis, and other clinical features between unilateral PTC patients with IC-LNM treated with uLT and TT.

## Patients and methods

### Patients

**Figure 1:**
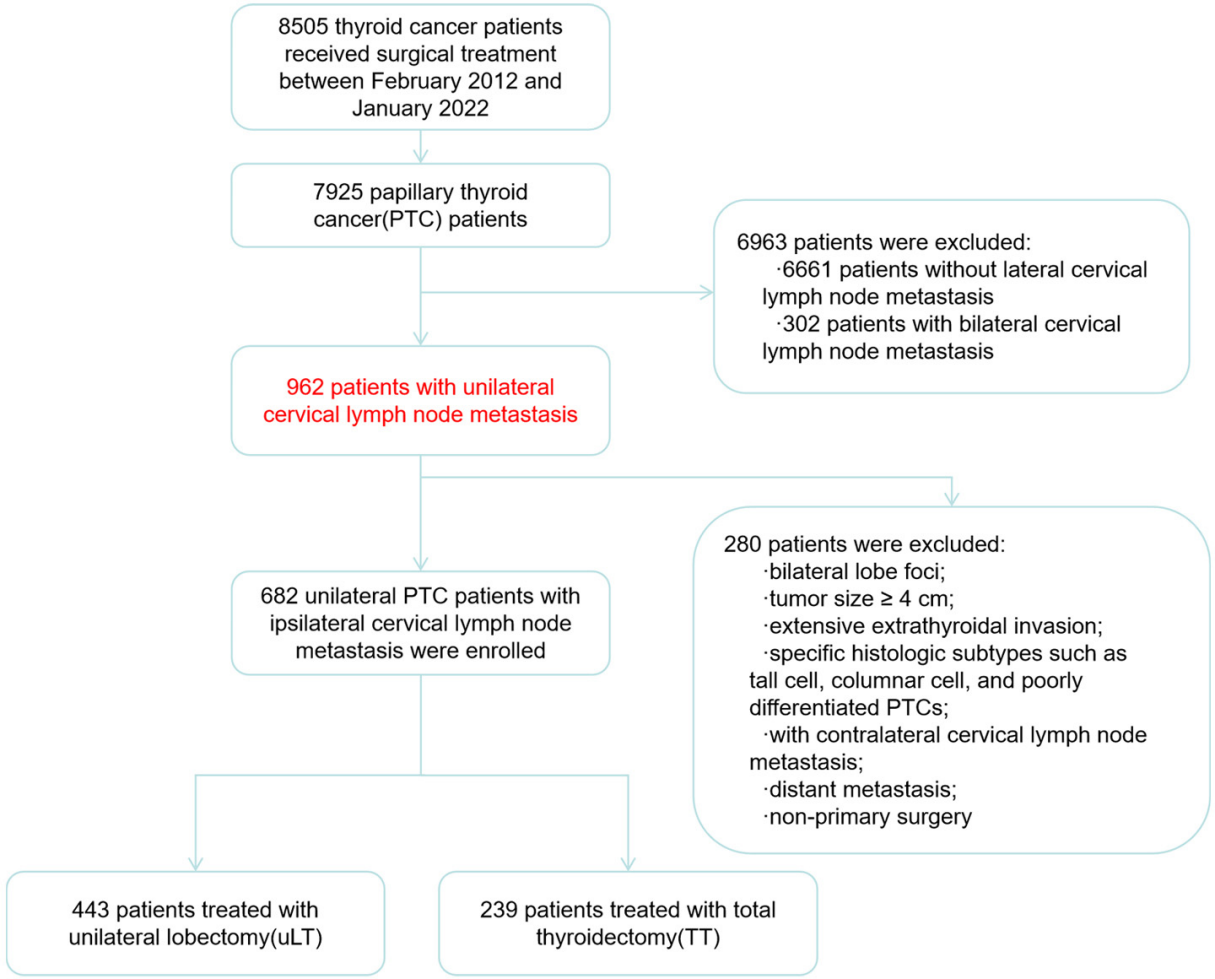
Flow chart of patient enrollment.

Thyroid cancer patients who received surgical treatment at Zhejiang Cancer Hospital from February 2012 to January 2022 were retrospectively analyzed (**Figure 1**). We collected clinical data including gender, age, surgery time, postoperative complications (such as superior laryngeal nerve injury, recurrent laryngeal nerve injury, transient or permanent hypoparathyroidism, infection of incisional wound, and postoperative hemorrhage, etc), hospital stays, hospitalization costs, recurrence-free survival (RFS), and overall survival (OS). Tumor stage and initial risk stratification were performed according to the American Joint Committee on Cancer (AJCC) 8th Edition TNM Staging System and the 2015 ATA guidelines. The inclusion criteria of patients were as follows: (1) pathologically confirmed unilateral PTC; (2) imaging suspected and fine-needle aspiration biopsy (FNAB) or intraoperative frozen section confirmed IC-LNM (level II-V); and (3) primary tumor treated with uLT (with or without isthmusectomy) or total (near-total) thyroidectomy and concurrent ipsilateral cervical lymph node dissection. Patients with any of the following characteristics were excluded: bilateral lobe foci; tumor size ≥ 4 cm; extensive extrathyroidal invasion (such as invasion of nerves, trachea, esophagus, prevertebral tissues;excluding micro-invading only the strap muscles); specific histologic subtypes such as tall cell, columnar cell, and poorly differentiated PTCs; with contralateral cervical lymph node metastasis; distant metastasis; non-primary surgery. A propensity score analysis with 1:1 matching based on the individual risk factors of gender, age, primary tumor size, multifocality, extrathyroidal invasion, and T stage was performed on uLT and TT groups. The study was approved by the Ethics Committee of Zhejiang Cancer Hospital.

### Outcomes and follow-up

The primary endpoints of this study were RFS and OS. RFS was defined as the interval from the date of initial surgery to the detection of locoregional recurrence or distant metastasis during follow-up, confirmed by imaging and either biopsy or postoperative pathological findings. OS was defined as the duration from the date of surgery to the date of death or the end of follow-up. Patients were routinely followed up at 1, 3, and 6 months postoperatively, and subsequently every 6 to 12 months, either through outpatient visits or telephone interviews. Specifically, at 1 month post-surgery, thyroid function and antibody levels—including thyroglobulin (Tg), anti-thyroglobulin antibody (TgAb), triiodothyronine (T3), thyroxine (T4), and thyroid-stimulating hormone (TSH)—were assessed. In subsequent follow-ups, in addition to these serological tests, all patients underwent thyroid and cervical lymph node ultrasound examinations. For patients with persistently elevated Tg or TgAb levels, further evaluation was performed using imaging studies, including neck ultrasound, and suspicious lymph nodes were subjected to FNAB and/or measurement of Tg in the aspirate (FNAB-Tg). If Tg or TgAb levels continued to rise without definite metastatic lesions on ultrasound, contrast-enhanced CT or MRI was recommended to identify potential local recurrence. In high-risk patients, chest CT was also performed to rule out distant metastases. For patients who have undergone TT, a whole-body radioactive iodine scan may be considered when there is a significant elevation in Tg or TgAb levels, despite the absence of evident lesions on ultrasound or CT imaging.

### Statistical analysis

Continuous variables were presented as the mean and standard deviation (SD), and tested with Student ‘s t-test; categorical variables were expressed as frequencies and proportions, and analyzed with Pearson’s χ2 test or Fisher’s exact test. RFS and OS were estimated using the Kaplan-Meier method, and intergroup curves were compared using the log rank test. Predictors of RFS were validated using univariate and multivariate Cox regression analyses, and hazard ratios (HRs) with 95% confidence intervals (CIs) were calculated. To control for potential confounders, individual patient propensity scores were calculated by logistic regression analysis. Next, propensity scores were used to pair uLT with TT groups in a 1:1 ratio with a tolerance of 0.02. The two-sided p-value <0.05 was considered statistically significant. All statistical analyses were performed using SPSS statistical software version 25.0 and Graphpad Prism.5.0.730.

## Results

### Baseline clinicopathological characteristics before and after propensity score matching

Ultimately, 682 unilateral PTC patients with IC-LNM were available in the study. Baseline characteristics of 682 unilateral PTC patients with IC-LNM according to surgical extent are listed in **Table 1**. A total of 443 patients treated with uLT and 239 patients underwent TT. There were no statistically significant differences in gender and age between the two groups. However, patients in the uLT group were more likely to have smaller tumors (<2 cm, 350 [79.0%]), earlier staging (stage I, 419[94.6%]), fewer numbers of lymph node metastases (median[range], 7[3-11] nodes), and fewer postoperative complication rates compared with the TT group (**Tables 1**, **2**).

**Table 1:** Baseline characteristics of 682 unilateral papillary thyroid carcinoma (PTC) patients with ipsilateral cervical lymph node metastasis.

Parameters	uLT (n=443)	TT (n=239)	p value
Age (years)			
<55	388 (87.6%)	197 (82.4%)	0.066
≥55	55 (12.4%)	42 (17.6%)	
Gender			
Male	174 (39.3%)	64 (46.0%)	0.157
Female	269 (60.7%)	175 (54.0%)	
Size (cm)			
<2	350 (79.0%)	165 (69.0%)	**0.004**
≥2	93 (21.0%)	74 (31.0%)	
Stage			
I	419 (94.6%)	213 (89.1%)	**0.009**
II-III	24 (5.4%)	26 (10.9%)	
T grade			
T1	338 (76.3%)	152 (63.6%)	**<0.001**
T2+T3	105 (23.7%)	87 (36.4%)	
Overall lymph node metastasis rate			
mean ± SD	0.24 ± 0.14	0.25 ± 0.15	0.109
Lateral lymph node metastasis rate			
mean ± SD	0.17 ± 0.12	0.19 ± 0.13	0.076
Number of lymph node metastasis			
mean ± SD	6.97 ± 4.34	8.39 ± 5.47	**<0.001**

uLT, unilateral lobectomy; TT, total thyroidectomy. Bold values, p < 0.05.

**Table 2:** Surgical complications according to surgical extent before and after PSM.

Parameters	Before PSM			After PSM		
	uLT (n=443)	TT (n=239)	p value	uLT (n=225)	TT (n=225)	p value
Transient hypoparathyroidism						
Absent	439 (99.1%)	147 (61.5%)	**<0.001**	221 (98.2%)	140 (62.2%)	**<0.001**
Present	4 (0.9%)	92 (38.5%)		4 (1.8%)	85 (37.8%)	
Permanent hypoparathyroidism						
Absent	443 (100%)	235 (98.3%)	**0.006**	225 (100%)	221 (98.2%)	**0.045**
Present	0(0%)	4(1.7%)		0(0%)	4(1.8%)	
Recurrent laryngeal nerve injury						
Absent	442 (99.8%)	239 (100%)	0.462	224 (99.6%)	225 (100%)	0.317
Present	1(0.2%)	0(0%)		1(0.4%)	0(0%)	

Hypoparathyroidism is characterized by hypocalcemia and low or undetectable levels of parathyroid hormone. Recurrent laryngeal nerve (RLN) injury is defined when the surgical records documented intraoperative RLN injury, or when the patients developed hoarseness after surgery, or when laryngoscopy revealed postoperative restriction of vocal cord movement.

uLT, unilateral lobectomy; TT, total thyroidectomy; PSM, propensity score matching. Bold values, p < 0.05.

After PSM with potential prognostic factors (such as gender, age, primary tumor size, multifocality, extrathyroidal invasion, and T stage), a total of 225 pairs of patients were included in the study. There were no statistically significant differences in the proportion of gender, age, primary tumor size, T stage, number of lymph node metastasis, lymph node metastasis rate, and TNM stage between the two groups (p>0.05; **Table 3**). However, postoperative complication rates were higher in the TT group than in the uLT group (93[41.3%] vs. 13[5.8%], p<0.001). Of the 225 patients in the TT group, 4 patients (1.8%, p=0.045) developed permanent hypoparathyroidism and 85 patients (37.8%, p<0.001) developed transient hypoparathyroidism (**Table 2**). All patients with transient hypoparathyroidism recovered within 6 months of healing.

**Table 3:** Baseline characteristics of 225 pairs unilateral PTC patients with ipsilateral cervical lymph node metastasis after propensity score matching (PSM).

Parameters	uLT (n=225)	TT (n=225)	p value
Age (years)			
<55	191 (84.9%)	186 (82.7%)	0.523
≥55	34 (15.1%)	39 (17.3%)	
Gender			
Male	65 (28.9%)	64 (28.4%)	0.917
Female	160 (71.1%)	161 (71.6%)	
Size (cm)			
<2	161 (71.6%)	162 (72.0%)	0.917
≥2	64 (28.4%)	63 (28.0%)	
Stage			
I	202 (89.8%)	202 (89.8%)	1.000
II-III	23 (10.2%)	23 (10.2%)	
T grade			
T1	144 (64.0%)	149 (66.2%)	0.621
T2+T3	81 (36.0%)	76 (33.8%)	
Overall lymph node metastasis rate			
mean ± SD	0.24 ± 0.14	0.25 ± 0.15	0.156
Lateral lymph node metastasis rate			
mean ± SD	0.18 ± 0.13	0.18 ± 0.13	0.500
Number of lymph node metastasis			
mean ± SD	4.32 ± 3.07	4.36 ± 3.08	0.481

uLT, unilateral lobectomy; TT, total thyroidectomy. Bold values, p < 0.05.

### Comparisons of time and financial factors in both uLT and TT groups

**Figure 2:**
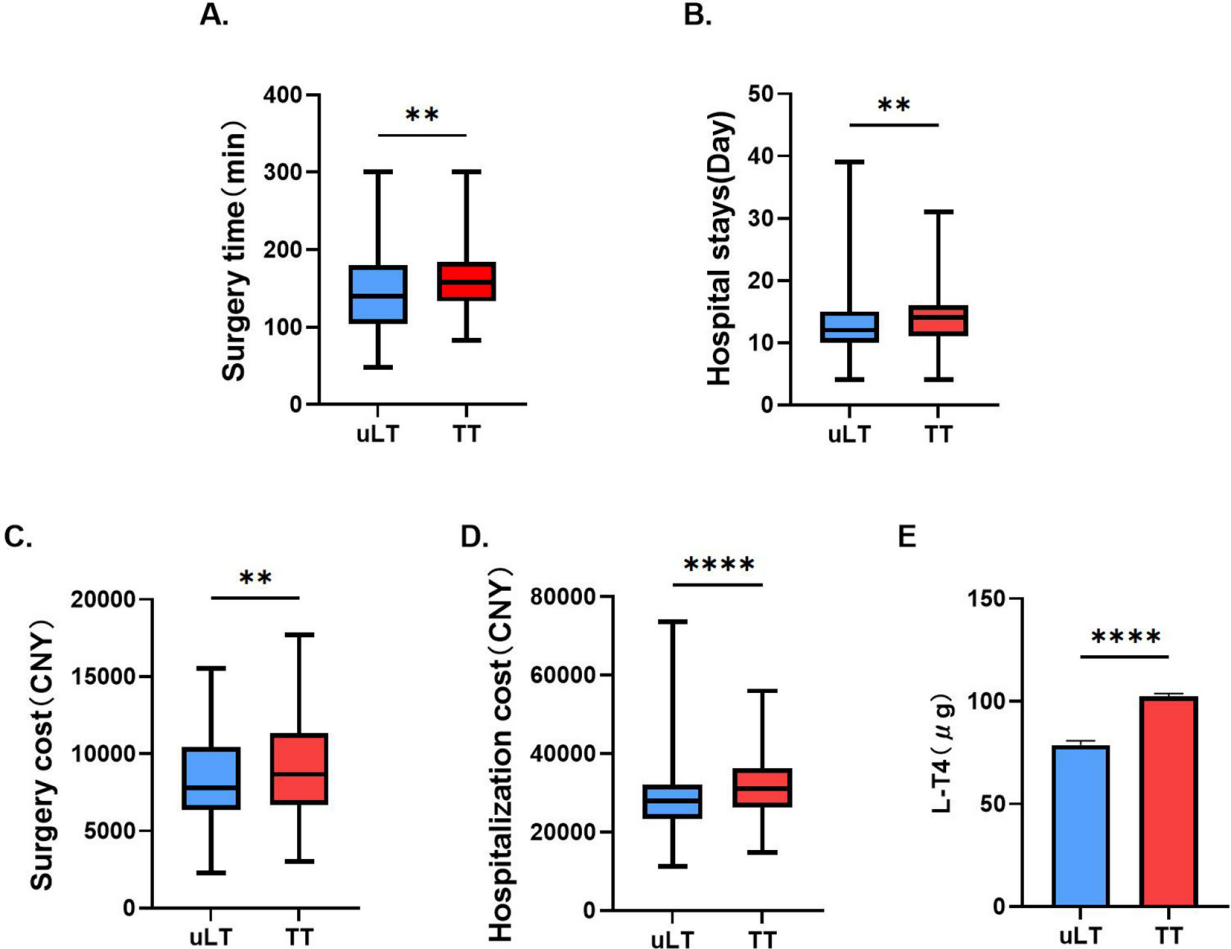
Comparison of surgery time **(A)**, hospital stays **(B)**, surgery cost **(C)**, hospitalization cost **(D)** and L-T4 doses at one month postoperatively **(E)** between unilateral lobectomy (uLT) and total thyroidectomy (TT) groups. **p< 0.01, ****p< 0.0001.

Patients treated with uLT spent less time on surgery (p<0.01) and hospitalization (p<0.01) compared to those treated with TT (**Figures 2A, B**). Moreover, we also found that this uLT group spent less money (p<0.01) on surgery and hospitalization (p<0.0001; **Figures 2C, D**). In addition, uLT reduced the L-T4 doses at one month postoperatively (**Figure 2E**). Thus, uLT is more cost-effective than TT, and these are equally important considerations for clinicians.

### Univariate and multivariate analyses of the risk factors of recurrence before and after PSM

**Tables 4**, **5** showed the risk factors of recurrence before and after PSM based on the univariate and multivariate analyses, respectively. Based on the univariate analysis, tumor size ≥2 cm, extrathyroidal extension, number of lymph node metastasis, overall lymph node metastasis rate, lateral lymph node metastasis rate and T2-3 stages were significant risk factors of recurrence before PSM, while tumor size ≥2 cm, number of lymph node metastasis, overall lymph node metastasis rate and T2-3 stages were significant risk factors of recurrence after PSM. However, based on the multivariate analysis, there were no significant predictors of recurrence before and after PSM (**Tables 4**, **5**).

**Table 4:** Univariate and multivariate analysis with Cox regression for all patients before PSM (n=682).

Parameters	Univariate			Multivariate		
	Hazard ratio (HR)	95% CI	p value	Hazard ratio (HR)	95% CI	p value
**Female gender**	0.723	0.407-1.284	0.269			
**Age(≥55 years)**	1.419	0.687-2.930	0.344			
**Total thyroidectomy**	0.710	0.380-1.325	0.282			
**Tumor size(≥2 cm)**	**2.269**	**1.278-4.028**	**0.005**	1.087	0.429-2.757	0.861
**Multifocality**	1.094	0.557-2.148	0.794			
**Extrathyroidal extension**	**1.920**	**0.999-3.690**	**0.050**	0.735	0.277-1.950	0.536
**Number of lymph node metastasis**	**1.190**	**1.100-1.286**	**<0.001**	1.107	0.966-1.268	0.143
**Overall lymph node metastasis rates**	**28.424**	**4.787-168.782**	**<0.001**	2.457	0.040-152.084	0.669
**Lateral lymph node metastasis rates**	**38.392**	**5.864-251.341**	**<0.001**	1.513	0.007-329.649	0.880
T						
T1	Ref			Ref		
T2-3	**2.833**	**1.608-4.991**	**<0.001**	2.798	0.943-8.299	0.064
TNM						
I	Ref					
II-III	1.957	0.876-4.370	0.102			

Ref, reference; CI, confidence interval. Bold values, p < 0.05.

**Table 5:** Univariate and multivariate analysis with Cox regression for all patients after PSM(n=225 pairs).

Parameters	Univariate			Multivariate		
	Hazard ratio (HR)	95% CI	p value	Hazard ratio (HR)	95% CI	p value
**Female gender**	0.654	0.327-1.306	0.228			
**Age(≥55 years)**	1.683	0.761-3.722	0.198			
**Total thyroidectomy**	0.570	0.282-1.152	0.118			
**Tumor size(≥2 cm)**	**2.203**	**1.118-4.338**	**0.022**	1.983	0.728-5.404	0.181
**Multifocality**	1.169	0.528-2.588	0.700			
**Extrathyroidal extension**	1.653	0.771-3.544	0.196			
**Number of lymph node metastasis**	**1.132**	**1.029-1.244**	**0.011**	1.072	0.925-1.243	0.355
**Overall lymph node metastasis rate**	**10.009**	**1.024-97.855**	**0.048**	1.906	0.076-47.649	0.695
**Lateral lymph node metastasis rate**	8.968	0.680-118.346	0.096			
T						
T1	Ref			Ref		
T2-3	**2.040**	**1.040-4.002**	**0.038**	1.374	0.506-3.729	0.533
TNM						
I	Ref					
II-III	1.814	0.751-4.383	0.186			

Ref, reference; CI, confidence interval. Bold values, p < 0.05.

### Survival analysis before and after PSM

**Figure 3:**
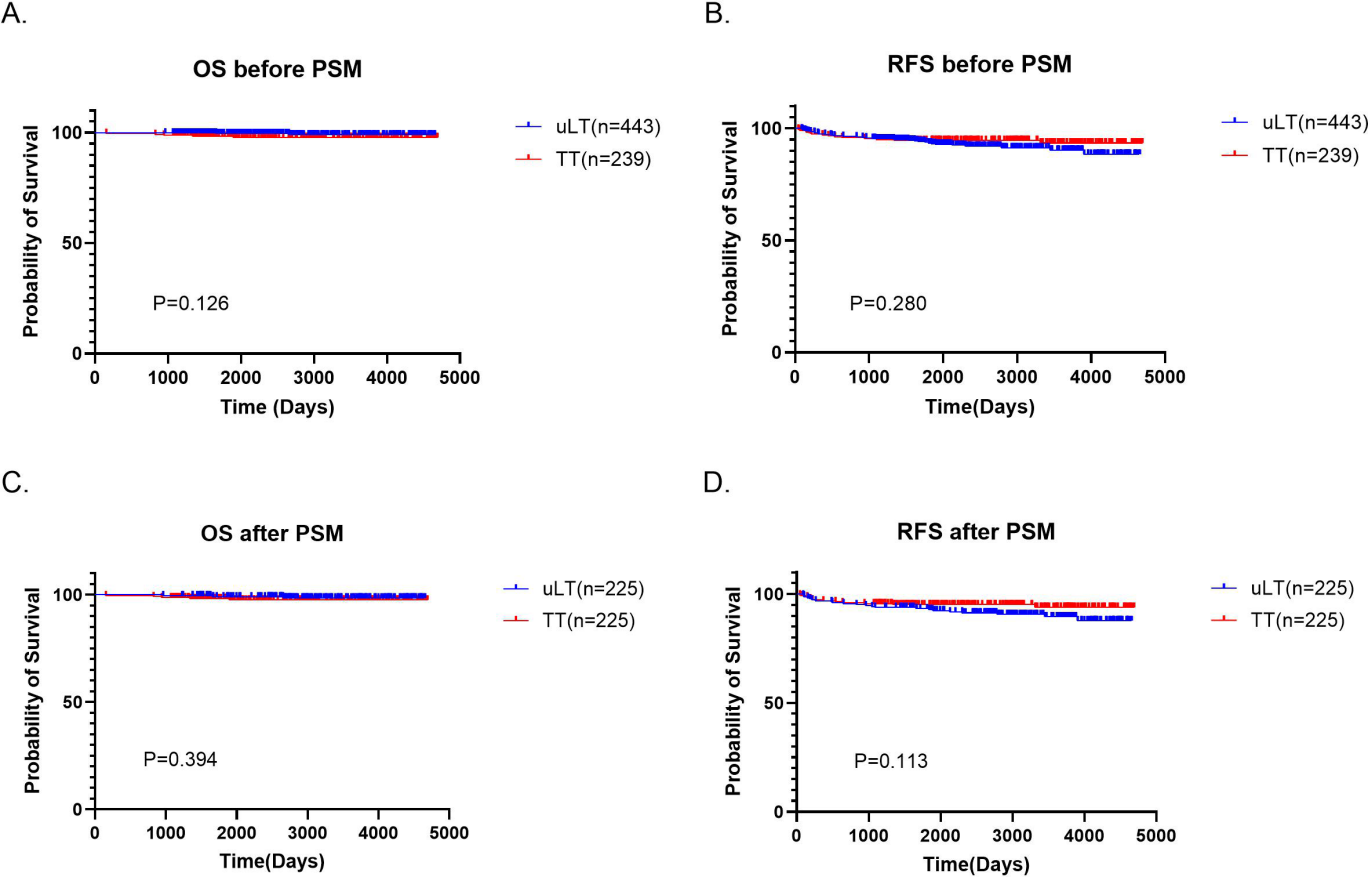
**(A, B)** Overall survival (OS) and recurrence-free survival (RFS) of uLT and TT groups before Propensity Score Matching (PSM). **(C, D)** OS and RFS of uLT and TT groups after PSM.

After a median follow-up of 81 (5-154) months, there were 3 deaths in the uLT group, none related to thyroid cancer. In the TT group, there were 5 deaths, of which two patients died of thyroid cancer. Five-year OS rates were 99.4% and 98.3% in the uLT group and TT group, respectively, and the differences were not statistically significant by the log-rank test (p=0.126; **Figure 3A**). Besides, 34 patients (7.7%) in the uLT group recurred, all of which were locoregional. These patients underwent a second surgery after recurrence, and 9 of them subsequently received postoperative postsurgical radioactive iodine (RAI) therapy, with favorable outcomes. In the TT group, 14 patients (5.9%) experienced recurrence, including one case of distant metastasis. All patients in this group also underwent reoperation, with 3 receiving additional RAI therapy postoperatively, and 2 patients ultimately died, while the remaining patients achieved stable disease control following treatment. Five-year RFS was 93.5% and 94.5% in the uLT group and TT group, respectively, and there were no statistically significant differences, either (p=0.280; **Figure 3B**). After PSM, the differences between uLT and TT groups in the 5-year OS (99.1% vs 98.2%, p=0.394) and 5-year RFS rates (93.3% vs 95.1%, p=0.113) were not statistically significant, either (**Figures 3C, D**).

**Figure 4:**
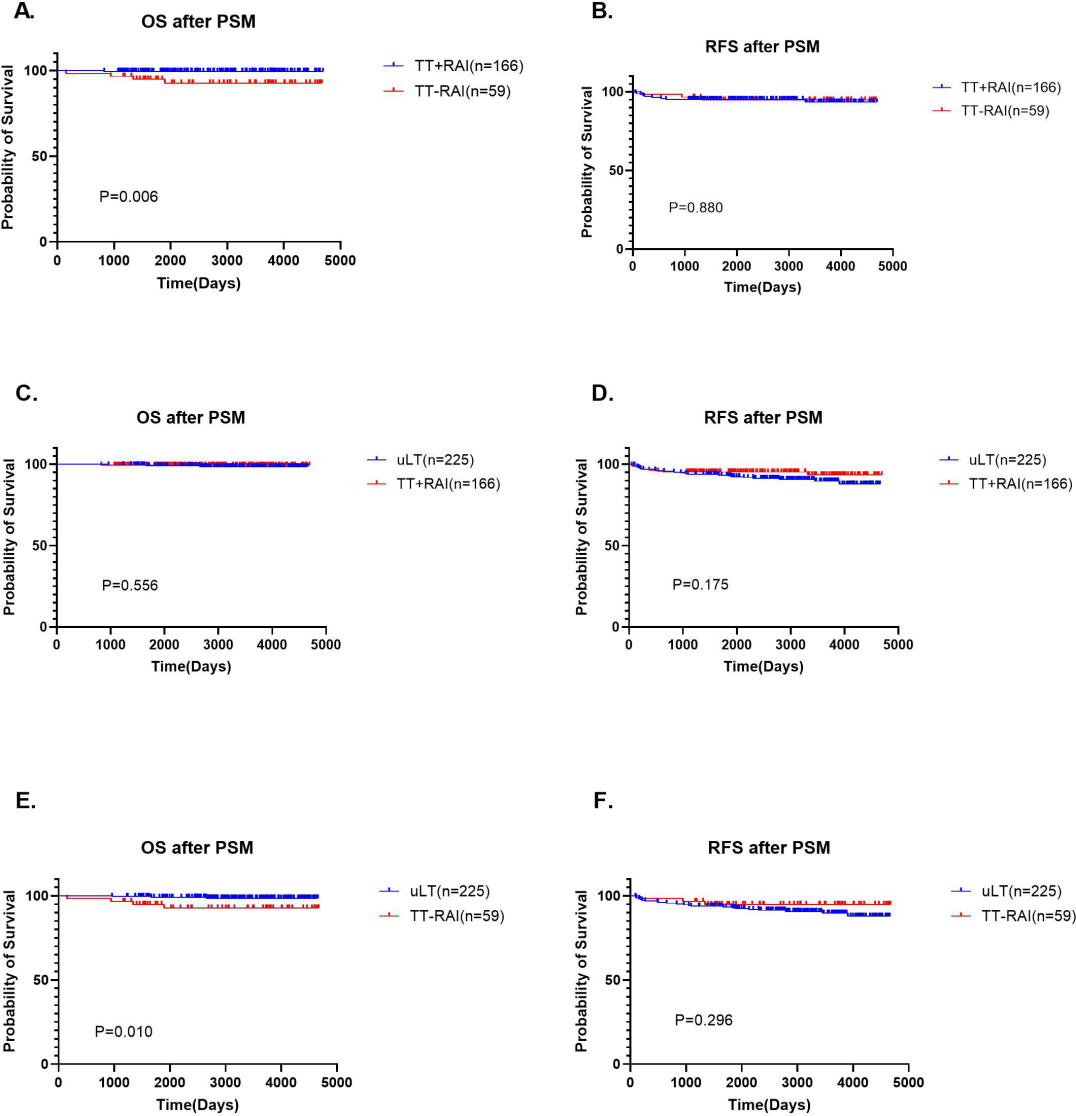
Kaplan-Meier plots for RFS and OS stratified by RAI therapy after PSM. **(A, B)** OS and RFS in TT treated with RAI therapy (TT+RAI) and TT untreated with RAI therapy (TT-RAI) groups. **(C, D)** OS and RFS in uLT and TT+RAI groups. **(E, F)** OS and RFS in uLT and TT-RAI groups.

We also verified the prognostic impact of postsurgical RAI therapy on TT. In the TT group, there were 166 patients who received RAI therapy (TT+RAIs) and 59 patients who did not receive RAI therapy (TT-RAIs), and the 5-year OS rates of TT+RAIs group was higher than TT-RAIs group (99.4% vs. 94.8%, p=0.006; **Figure 4A**). However, there was no significant difference in 5-year RFS (95.2% vs 94.8%, p=0.880; **Figure 4B**). Due to the differences in 5-year OS, we performed univariate and multivariate analyses of the risk factors of death on these 166 TT+RAIs and 59 TT-RAIs (**Supplementary Table 1**). The results showed that RAI therapy after TT might reduce the risk of death. Stratified analyses were performed on patients in the uLT group (n=225) and the TT+RAI group (n=166), and there were no statistically significant differences in 5-year OS rates (99.1% vs. 99.4%, p=0.556) and 5-year RFS rates (93.3% vs. 95.2%, p=0.175) between the two groups (**Figures 4C, D**). After excluding patients who received RAI therapy, the 5-year OS rates in the uLT group (n=225) was higher than that in the TT-RAIs group (n=59) (99.1% vs. 94.8%, p=0.010; **Figure 4E**). However, the 5-year RFS rates in the uLT group (n=225) and TT-RAIs group (n=59) were 93.3% and 94.8% (p=0.296), respectively, and the differences were not statistically significant (**Figure 4F**). Univariate and multivariate analyses results indicated no significant predictors of survival (**Supplementary Table 2**).

## Discussion

Most PTC patients have a favorable prognosis. Precise stratification of treatment according to the different levels of risk for PTC can help to better reduce the risk of complications associated with surgery and other treatments while improving the long-term survival of the patients, thereby improving the quality of life of the patients and reducing the socio-economic burden (21, 22). A growing number of studies have shown that patients undergoing TT have no significant survival advantages over patients undergoing lobectomy, especially for 1-4 cm tumors, with a low- to intermediate-risk of recurrence (10, 23). Adam et al. evaluated the National Cancer Database data of 61775 PTC patients, and the results showed that TT had no survival advantage compared with lobectomy (9). The 2015 ATA guidelines have made some changes to the surgical treatment of PTC, with the option of lobectomy or TT for 1-4 cm PTC without high-risk features (16). However, it remains to be explored which surgery is optimal for PTC patients with IC-LNM.

In this retrospective cohort study, we compared the differences in risk and benefit between unilateral PTC patients with IC-LNM in treatment with uLT and TT in terms of postoperative complications, prognosis, total hospitalization cost, etc. To minimize the selection bias, we performed PSM analysis to correct for confounding factors such as tumor stage and size. In the pre-matched and post-matched outcomes, TT and uLT group had similar recurrence and survival, while TT group had a higher risk of complications, including hypoparathyroidism, which is consistent with previous studies (23–25). In addition, we found that uLT had a lower surgical cost and shorter hospital stays than TT. For uLT patients, the production of thyroid hormones by the contralateral thyroid gland could be preserved, and the doses and side effects of postoperative L-T4 intake might be reduced. Therefore, considering that differentiated thyroid carcinoma patients under treatment wish to not only improve survival and reduce recurrence/persistence, but also improve postoperative quality of life, as well as reduce the socioeconomic burden, uLT might be a more economical, efficient, and patient-friendly surgery.

For patients undergoing TT, RAI therapy can be performed after surgery, thus facilitating the use of Tg as a sensitive tumor marker for residual thyroid tissue or recurrence during follow-up (26–28). In our study, after PSM for major risk factors to minimize selection bias, 59 patients undergoing TT (26.2%) were not treated with RAI postoperatively, and 4 of these patients died. The cause of 2 of these patients’ deaths were related to thyroid cancer, due to the occurrence of tumor metastasis. There were no significant differences in prognosis between TT+RAI and uLT groups; after excluding patients with TT+RAI, the OS was worse in the TT group than in the uLT group (p=0.010). Our studies demonstrated that patients not received RAI therapy after TT might affect their prognosis, while the prognosis of patients who received RAI were not statistically significantly different from that of patients with uLT. These results showed the survival impact of RAI therapy on TT. In other words, TT treatment did not prevail compared with uLT, and the impact of RAI therapy should be taken into account, especially for those older age people (≥55 years). However, the effect of RAI therapy in improving prognosis remains controversial. Referring to the ATA guidelines for recurrence risk stratification, postoperative RAI therapy would be recommended in both the intermediate- and high- risk groups. RAI therapy in the low-risk group is controversial for patient prognosis and is not routinely recommended (16). Seo et al. showed that postoperative RAI therapy did not significantly reduce the risk of recurrence in intermediate-risk PTC patients, PTC patients should be considered on the basis of specific risk re-stratification (29). Youngmin et al. speculated that RAI therapy might be ineffective in reducing the recurrence of multifocal PTC patients (30). RAI refractoriness occurs in some PTC patients and dramatically decreased RAI uptake, which affects prognosis (31, 32). In addition, preparation for RAI therapy requires discontinuation of thyroid hormone or recombinant TSH and a low iodine diet, which reduces quality of life, and the risk of complications associated with repeated iodine-131 treatments with high iodine uptake, such as xerostomia, recurrent sialoadenitis and pulmonary fibrosis, also need to be taken into account (33, 34). All in all, available studies have not been able to assess the impact of RAI therapy on clinical outcomes in patients treated with TT.

On the one hand, previous studies were limited to small numbers of patients, excluding adequate multivariate adjustment. In our study, the larger number of patients enabled us to perform PSM for possible prognostic factors in PTC, which could minimize the selection bias. Prospective studies are challenging to implement since most PTCs have a favorable prognosis. On the other hand, the current study also had limitations. Our study was a single-center retrospective study, might limit the conclusions. So that further large-sample and multicenter studies were needed for validation. Besides, while serum Tg levels can be effectively used for early detection of recurrence in patients who have undergone TT, this method is not applicable to those who underwent uLT, as residual thyroid tissue compromises the reliability of Tg-based monitoring.

In conclusion, this study showed that the uLT group and TT group presented similar prognostic outcomes, while the uLT group had a lower risk of postoperative complications and lower hospitalization costs than the TT group. Therefore, for reasonably selected unilateral PTC patients without other high-risk features (such as bilateral lobe foci; tumor size ≥ 4 cm; extensive extrathyroidal invasion; with contralateral cervical lymph node metastasis; distant metastasis), uLT could be recommended with IC-LNM.

## Data Availability

The raw data supporting the conclusions of this article will be made available by the authors, without undue reservation.
