# Supplementary material for: Lobectomy vs total thyroidectomy for unilateral papillary thyroid carcinoma with ipsilateral cervical lymph node metastasis
Source: Front Endocrinol (Lausanne). 2025 Jul 24;16:1564752. doi: 10.3389/fendo.2025.1564752 (PMC12328177; doi:10.3389/fendo.2025.1564752)
Supplement: Supplementary file 1 [file Table1.docx]

**Supplementary Table 1.** Univariate and multivariate analysis with Cox regression for 166 patients who received RAI ablation(TT+RAIs) and 59 patients who did not receive RAI ablation(TT-RAIs) in TT group

|  | **Univariate** | | |  | **Multivariate** | | |
| --- | --- | --- | --- | --- | --- | --- | --- |
|  | **Hazard ratio (HR)** | **95% CI** | **p value** |  | **Hazard ratio (HR)** | **95% CI** | **p value** |
| **Female gender** | 1.589 | 0.178-14.219 | 0.679 |  |  |  |  |
| **Age (≥55 years)** | **7.253** | **1.212-43.420** | **0.030** |  | 5.520 | 0.912-33.418 | 0.063 |
| **RAI ablation** | **0.088** | **0.010-0.792** | **0.030** |  | **0.108** | **0.012-0.984** | **0.048** |
| **Tumor size (≥2 cm)** | 0.664 | 0.074-5.942 | 0.714 |  |  |  |  |
| **Multifocality** | 2.665 | 0.445-15.967 | 0.283 |  |  |  |  |
| **Extrathyroidal extension** | 1.059 | 0.118-9.475 | 0.959 |  |  |  |  |
| **Number of lymph node metastasis** | 0.944 | 0.693-1.285 | 0.714 |  |  |  |  |
| **T** |  |  |  |  |  |  |  |
| T1 | Ref | | |  |  | | |
| T2-3 | 0.495 | 0.055-4.426 | 0.529 |  |  |  |  |
| **TNM** |  |  |  |  |  |  |  |
| I | Ref | | |  |  | | |
| II-III | 5.483 | 0.915-32.868 | 0.063 |  |  |  |  |

Abbreviations: Ref, reference; CI, confidence interval
