# Supplementary material for: Lobectomy vs total thyroidectomy for unilateral papillary thyroid carcinoma with ipsilateral cervical lymph node metastasis
Source: Front Endocrinol (Lausanne). 2025 Jul 24;16:1564752. doi: 10.3389/fendo.2025.1564752 (PMC12328177; doi:10.3389/fendo.2025.1564752)
Supplement: Supplementary file 2 [file Table2.docx]

**Supplementary Table 2.** Univariate and multivariate analysis with Cox regression for 284 patients who did not receive RAI ablation

|  | **Univariate** | | |  | **Multivariate** | | |
| --- | --- | --- | --- | --- | --- | --- | --- |
|  | **Hazard ratio (HR)** | **95% CI** | **p value** |  | **Hazard ratio (HR)** | **95% CI** | **p value** |
| **Female gender** | 0.496 | 0.111-2.217 | 0.358 |  |  |  |  |
| **Age (≥55 years)** | **14.023** | **2.711-72.534** | **0.002** |  | 7.739 | 0.701-85.504 | 0.095 |
| **Total thyroidectomy** | **5.669** | **1.267-25.368** | **0.023** |  | 4.070 | 0.896-18.491 | 0.069 |
| **Tumor size (≥2 cm)** | 0.480 | 0.058-3.994 | 0.497 |  |  |  |  |
| **Multifocality** | 1.637 | 0.317-8.443 | 0.556 |  |  |  |  |
| **Extrathyroidal extension** | 2.140 | 0.414-11.062 | 0.364 |  |  |  |  |
| **Number of lymph node metastasis** | 1.101 | 0.890-1.362 | 0.376 |  |  |  |  |
| **T** |  |  |  |  |  |  |  |
| T1 | Ref | | |  |  | | |
| T2-3 | 0.870 | 0.168-4.497 | 0.868 |  |  |  |  |
| **TNM** |  |  |  |  |  |  |  |
| I | Ref | | |  | Ref | | |
| II-III | **11.107** | **2.484-49.653** | **0.002** |  | 1.721 | 0.192-15.416 | 0.628 |

Abbreviations: Ref, reference; CI, confidence interval
